# Chemogenomics approaches to rationalising compound action of traditional Chinese and Ayurvedic medicines

**DOI:** 10.1186/1758-2946-5-S1-P44

**Published:** 2013-03-22

**Authors:** Fazlin Mohd Fauzi, Alexios Koutsoukas, Rob Lowe, Kalpana Joshi, Tai-Ping Fan, Andreas Bender

**Affiliations:** 1 Unilever Centre for Molecular Science Informatics, Department of Chemistry, University of Cambridge, Lensfield Road, CB2 1EW, UK; 2 Universiti Teknologi MARA (UiTM) Malaysia, 40 450 Shah Alam, Selangor, Malaysia; 3 Blizard Institute of Cell and Molecular Science, Barts and The London School of Medicine and Dentistry, The Blizard Building, 4 Newark Street, London E1 2AT, UK; 4 Symbiosis School of Biomedical Sciences, Symbiosis International University, Pune, India; 5 Department of Pharmacology, University of Cambridge, Tennis Court Road, Cambridge, CB2 1PD, UK

## 

Traditional Chinese medicine (TCM) and Ayurveda have been used in humans for thousands of years [1]. While the link to a particular indication has been established in man, the mode-of-action (MOA) of the formulations is relatively unknown. In this study, we aim to understand the MOA of formulations used in traditional medicine using *in silico *target prediction tools, which predicts protein targets (hence, MOAs) given the chemical structure of a compound. We were able to establish several links between suggested MOAs and experimental evidence. In particular, compounds from the 'tonifying and replenishing medicinal' class exhibit a hypoglycemic effect [2] which can be connected to SGLT 1 and 2 [3] and PTP1B [4]. Similar results were obtained with Ayurvedic anti-cancer drugs. Here, both primary anti-cancer targets, which directly participate in cancer pathogenesis, *i.e*. steroid-5-alpha-reductase 1 and 2 were predicted, as well as synergistic targets, *i.e*. P-glycoprotein (blocking this efflux pump increases intracellular concentration of the primary active ingredient) [5]. In addition, some targets may point us to possible novel MOA and side effects. Most notably, GPBAR1 which was predicted as a target for both 'tonifying and replenishing medicinal' and anti-cancer classes, suggest an influence of the compounds on metabolism [6]. Understanding the MOA of these compounds is beneficial as it can provide new resources for NME, with higher efficacies in the clinic than in the current drug discovery setting. This can be a promising endeavor as the phenotypes of these compounds are well known which indicates both the therapeutic impact and efficacy against a certain disease.
